# Influence of bone-conducted vibration on simulator sickness in virtual reality

**DOI:** 10.1371/journal.pone.0194137

**Published:** 2018-03-28

**Authors:** Séamas Weech, Jae Moon, Nikolaus F. Troje

**Affiliations:** 1 Department of Psychology, Queen’s University, Kingston, Ontario, Canada; 2 Centre for Neuroscience Studies, Queen’s University, Kingston, Ontario, Canada; 3 Department of Biology, Queen’s University, Kingston, Ontario, Canada; 4 School of Computing, Queen’s University, Kingston, Ontario, Canada; University of Minnesota, UNITED STATES

## Abstract

Use of virtual reality (VR) technology is often accompanied by a series of unwanted symptoms, including nausea and headache, which are characterised as ‘simulator sickness’. Sensory mismatch has been thought to lie at the heart of the problem and recent studies have shown that reducing cue mismatch in VR can have a therapeutic effect. Specifically, electrical stimulation of vestibular afferent nerves (galvanic vestibular stimulation; GVS) can reduce simulator sickness in VR. However, GVS poses a risk to certain populations and can also result in negative symptoms in normal, healthy individuals. Here, we tested whether noisy vestibular stimulation through bone-vibration can also reduce symptoms of simulator sickness. We carried out two experiments in which participants performed a spatial navigation task in VR and completed the Simulator Sickness Questionnaire over a series of trials. Experiment 1 was conducted using a high-end projection-based VR display, whereas Experiment 2 involved the use of a consumer head mounted display. During each trial, vestibular stimulation was either: 1) absent; 2) coupled with large angular accelerations of the projection camera; or 3) applied randomly throughout each trial. In half of the trials, participants actively navigated using a motion controller, and in the other half they were moved passively through the environment along pre-recorded motion trajectories. In both experiments we obtained lower simulator sickness scores when vestibular stimulation was coupled with angular accelerations of the camera. This effect was obtained for both active and passive movement control conditions, which did not differ. The results suggest that noisy vestibular stimulation can reduce simulator sickness, and that this effect appears to generalize across VR conditions. We propose further examination of this stimulation technique.

## Introduction

Recently, technological advances have supported a proliferation of inexpensive and powerful consumer-oriented virtual reality (VR) hardware devices. This advancement creates an urgent need to solve some of the key problems of VR exposure. Perhaps the principle problem is a phenomenon known as ‘simulator sickness’ (also known as ‘cybersickness’ [1–2]). Around 80% of VR users typically experience some symptoms of sickness, with as many as 50% experiencing symptoms with such severity that they are compelled to terminate a session of VR early [3]. The most common adverse effects of virtual environment immersion include nausea, headache, sweating, and vomiting. These symptoms can persist for several hours following exposure to the environment [4–5]. The symptoms are often sufficient to compel users to avoid further use of VR entirely [6–7]. Given that VR technology offers a valuable method for use in skills training, education, and clinical rehabilitation, there has been a substantial amount of research into the causes of simulator sickness in VR [3].

### Causes of simulator sickness

A number of contributing factors have been implicated in the etiology of simulator sickness, including visual flicker, low refresh-rate, and high motion-to-photon latency [6,8–10]. As tracking and display technology continues to develop, user comfort is expected to increase—although some display improvements may in fact exacerbate symptoms, such as increases in the field-of-view [11]. Frequently, VR experiences simulate self-motion through an environment using optic flow, and this manner of simulation appears to be a particular trigger for sickness [10–13]. It is well known that optic flow is sufficient to specify motion of an observer through their environment [14–15]. However, if the vestibular sense does not receive stimulation at the moment of motion onset and offset to indicate body accelerations, sensory information is incongruent. Symptoms are thought to occur as a result of the nervous system attempting to respond appropriately to sensory mismatch, which is a situation that might have been caused internally (e.g., as the results of accidental ingestion of a neurotoxic substance [13,16–17]). In that case, nausea and the emptying of the stomach could be considered an adaptive function, although the response becomes severely maladaptive when the sensory conflicts result from curve navigation in a driving simulator. Another explanation for simulator sickness has focused on the postural instability produced by exposure to VR technology [18–19]. It is possible that decreased postural stability in VR increases the number and magnitude of cue-conflicts that may underlie symptoms of discomfort, although empirical evidence is as of yet unclear [20–21].

### Techniques for reducing simulator sickness

Despite the understanding acquired about the causes of simulator sickness, its prevention and treatment have received less attention. One preventative approach has been to avoid situations that generate sensory mismatch: For example, Dorado and Figueroa [22] implemented camera movement in VR that avoids accelerations as much as possible. They showed that using ramps instead of staircases for changing elevation in the environment can reduce the degree of simulator sickness experience by the user. Recently a ‘point and teleport’ method for moving in a virtual world has gained popularity, where a user specifies a position to which they will relocate upon a button press [23]–this technique also minimizes the accelerations of the visual scene. Another method has focused on preventing sensory mismatch by ‘recoupling’ the visual and vestibular systems during navigation of a VR environment. Some approaches involve using motion platforms to move the body along with visually-simulated motion [24–25], and several consumer-oriented motion simulators are beginning to emerge. The efficacy of motion base simulators in reducing simulator sickness is not clear, however. While studies show improved comfort for moving base compared to fixed base simulators [26–27], others indicate no effect of motion cueing [28], or even an exacerbating effect on symptom severity [29]. Compared to their significant expense and technical complexity, the current balance of research shows little evidence that motion platforms effectively reduce symptom severity compared to stationary conditions [9,30–31]. Another technique, galvanic vestibular stimulation (GVS), has been effective in preventing symptoms of simulator sickness in virtual environments. This technique involves applying an electrical current to electrodes near the mastoid processes in order to stimulate vestibular afferent nerves. Applying GVS to recouple visual and vestibular cues was shown to reduce the incidence of simulator sickness in a flight simulator task [32]. The technique has found additional support in a study by Reed-Jones and colleagues [33], where simulator sickness was reduced by using GVS in a driving simulator. This is also supported by additional evidence, showing a preventive effect of galvanic stimulation on simulator sickness in a driving task, regardless of whether stimulation is applied during curve maneuvers or intermittently throughout the task [34].

The visual-vestibular recoupling approach to simulator sickness has led to the development of preliminary consumer-oriented GVS devices [35]. Nonetheless, a series of practical issues remain in terms of the use of GVS in VR experiences. Previous research indicates that GVS use is associated with symptoms of discomfort in some healthy users [36]. For certain individuals, such as pacemaker users, there are serious risks involved in applying direct current stimulation to the surface of the body, as is the case with GVS [37]. An additional obstacle to the widespread adoption of GVS is the precise match between vision and vestibular stimulation required in order to accurately replace the expected vestibular signals. Small errors between directional cues derived from vision and those that are applied using GVS could engender sensory mismatches that impact performance and comfort significantly [37].

Recent research from our group has employed a vestibular stimulation method that presents a possible solution to both the problem of invasiveness and the problem of precision described above. The method we have used involves applying noisy stimulation to the vestibular system using bone-conducted vibration (BCV) that is applied at the mastoid processes. This technique has been shown to evoke the oculomotor and myogenic responses similar to those produced by linear accelerations of the otolith organs [38–42]. Unlike with GVS, there are no known populations for whom BCV produces adverse effects, according to evidence obtained with well over 3000 participants [43]. At the same time, we contend that the use of BCV to reduce sensory mismatch does not require a precise mapping between the expected vestibular signal and the applied vestibular signal, given that the intention of the approach is to add sensory noise to the vestibular system. This reduced constraint therefore renders BCV easier to implement than GVS, where the aim is typically to ‘recouple’ vision and vestibular cues [32–34]. In addition, we recently proposed that BCV reduces the sensory reliability of the vestibular system, which has the consequence of upweighting visual self-motion information that is obtained during stimulation. This theory was presented on the basis of evidence that visually evoked illusions of self-motion (vection) are facilitated by noisy stimulation of the vestibular system with both BCV and noisy GVS [44]. The idea builds on a Bayesian cue integration framework where sensory cues are inversely weighted by their reliability [45–47].

The results of previous work conducted by our group [44] provided strong evidence that BCV–an otolith stimulation [38, 43]–facilitates quicker vection when it is applied in conditions in which no otolith stimulation would be expected (e.g., yaw rotation about the vertical axis). This finding points towards a general effect of BCV on vestibular processing, which we attributed to a reduction in vestibular reliability. The same study also disputes the possibility that noisy vestibular stimulation simply masks the input to vestibular organs, since we observed similar effects between BCV (otolith) and noisy GVS (non-specific vestibular afferent stimulation [48]). In the context of the relationship between sensory conflict and simulator sickness proposed by Reason and Brand [13], we expected that reducing vestibular reliability in this manner would give rise to reduced conflict and improved comfort in VR. We designed the current study to test this possibility.

### Study aims

The aim of the current study was to employ BCV as a novel technique for reducing simulator sickness. We tested the effect of two versions of BCV on simulator sickness while participants completed a path navigation task which was rich in simulated self-motion. We coupled the timing of BCV stimulation to visual angular accelerations in one condition, and in the other condition we applied BCV at random intervals.

Our main prediction was that when BCV is applied during large visual accelerations (that is, when significant vestibular cues would normally be expected to occur), simulator sickness will be reduced compared to control conditions. We reasoned that the absence of vestibular motion cues accompanying large visual acceleration contributes significantly to simulator sickness, as has been proposed by several previous studies [10–13]. Applying BCV along with large visual accelerations should therefore encourage visual self-motion cues to be upweighted against the noisy and therefore unreliable vestibular input. On the other hand, BCV that is applied randomly throughout a trial should have little effect on simulator sickness scores as it is not directly associated with visual acceleration cues. While linear acceleration of the head and tilt with respect to gravity are also adequate vestibular cues, we only coupled the timing of BCV stimulation to angular accelerations of the camera in the present study. If BCV causes a general reduction in vestibular reliability, coupling BCV to any adequate vestibular stimuli should produce similar results. However, given that coupling the timing of BCV to linear accelerations or tilt would have resulted in a near-constant vibration, and for the purposes of time, we decided to test BCV coupled only to the timing of angular acceleration.

In addition to our main objective, there were two secondary objectives of the current study. First, we aimed to test if BCV reduced simulator sickness in both active and passive movement control conditions; that is, when participants control their own movement in the VR environment, and when they move passively through the environment. The degree of movement-control participants exert in VR is typically related to measures of simulator sickness [49–51]. We were mainly interested in this factor because of the prevalence of passive simulated self-motion in consumer-oriented VR experiences [23]. Second, we wanted to assess if the typical linear increase in symptom severity observed over time during VR exposure [52–54] would be affected by BCV stimulation.

In Experiment 1, we designed a VR navigation task to test the effect of BCV on simulator sickness. Given the proposed link between simulator sickness and errors in visual and vestibular self-motion estimates [12–13], we designed a spatial navigation task that involved simulated observer motion. We used a high-end projection-based VR system with motion tracking to present the task. Across three groups we either: 1) applied BCV when visual flow implied angular accelerations greater than 3 deg/s^2^, 2) applied BCV randomly throughout the trial, or 3) applied no stimulation. In all conditions, participants conducted both active trials (participant controlled the movement) and passive trials (automatic movement). After each trial we measured simulator sickness using the SSQ. We were interested in an overall effect of stimulation on SSQ scores, but we also wanted to assess if the increase in simulator sickness over a series of trials would differ for the participants who received BCV stimulation.

In Experiment 2, we closely replicated the task conditions of Experiment 1 with an off-the-shelf head-mounted display. Our aim was to characterize the degree to which noisy vestibular stimulation is effective at preventing simulator sickness across different VR display technologies.

## Ethics statement

The Queen’s University General Research Ethics Board (GREB) approved this research and all methods were in accordance with the Declaration of Helsinki. Upon arriving at the lab, each participant provided verbal and written informed consent before completing any questionnaires. At the end of the experiment, participants were verbally debriefed and given a written debriefing form complete with contact information for the Queen’s University GREB. In line with the Queen’s University GREB and Canadian federal law, we did not require parental consent from participants who were under the age of 18 at the time of their participation in this study, as post-secondary students are considered able to provide their own consent in Canada. The Queen’s University GREB approved this consent procedure. All relevant variables and analyses conducted on the data are reported in the article.

## Experiment 1

### Methods

#### Participants

Participants were recruited from a student mailing list at Queen’s University. A-priori, we chose a desired sample size of thirty participants and elected to replace participants who could not complete the experiment until this sample size was met. Thirty participants (22 women) completed the experiment. Four participants terminated the experiment early due to a high level of simulator sickness, and their data were not included in the final analyses. Each participant received $10 per hour. Mean age was 19.80 years (*SD* = 2.46, range = [18, 27]). All participants had normal or corrected to normal vision.

Participants were asked the following question prior to attending the study: “In daily life, how likely are you to experience motion sickness? (e.g., when traveling in a car or plane)”. Responses were given by indicating a point on a scale from 0 to 10 with anchors of “Not at all likely” and “Extremely likely”. Those who marked 9 or 10 would have been advised not to take part in the study as we considered it likely that such participants would experience severe discomfort in the experiment. However, we obtained no responses above 8 in this study.

#### Vestibular stimulus

We secured bone vibrators (Radioear B-71, New Eagle, PA) to the left and right mastoid processes using an elasticated head-band. The voltage signal used to drive the vibrators was delivered using a sound card attached to a custom-built audio amplifier.

There is a well-defined frequency tuning range for BCV: vibration between 200 and 500 Hz produces the largest myogenic potentials [40]. In our experiments, the vibrators operated at a frequency of 500 Hz. Each burst of stimulation lasted 250 ms. We selected a standard BCV magnitude based on the magnitude of stimulation that produced an effect on self-motion perception in a previous study we conducted on self-motion perception [44]. If the intensity of the BCV stimulation was uncomfortable for the participant, we reduced it incrementally until it reached the level that the participant verbally rated as ‘tolerable’. This was important given that the vibration magnitude at the level of the bone depends on a variety of factors, including the shape and size of the head of the participant [38–39].

#### Visual stimulus

**Fig 1:**
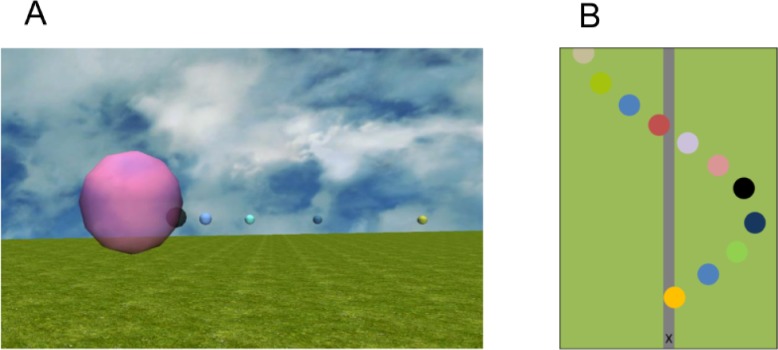
Virtual environment in Experiment 1. A) Detail of the virtual environment seen by participants. B) Top-down view of the initial section of the path. Participants started each trial at the X. (Targets are scaled up 10 times in size to aid visibility).

We created the task and visual stimulus in Vizard (Version 5.0, WorldViz LLC, Santa Barbara, CA) using the Python programming language (version 2.7). The ground plane was textured with grass (dimensions: 350 x 100 metres). An airport runway was positioned in the centre of the ground plane (dimensions: 350 x 5 metres) to act as a reference frame for participants. We generated a path for participants to navigate by positioning 30 spherical targets in the environment (Fig 1). The targets were coloured randomly, each had a diameter of 1 metre, and each was positioned 3 metres above the ground plane. The path consisted of two lateral cycles of a sine wave (dimensions: 315 x 80 metres, the formula for the path can be specified as: *y* = 40 sin(2 π *x*/157.5), where *y* is left-right and *x* is fore-aft).

Participants navigated their way through the virtual environment using a handheld controller (Flystick3, Advanced Realtime Tracking, Weilheim i.OB, Germany) that was tracked by an optical motion tracking system. The projection camera maintained a constant velocity of 5.5 m/s in the direction of heading, and the heading direction was controlled by the orientation of the handheld controller. The rotation angle of the controller in pitch, roll, and yaw measured in degrees in world coordinates directly specified the angular velocity of the camera (measured in degrees per second) in each axis. For example, if the controller was held at an angle of 10 deg in pitch, the angular velocity of the camera in pitch was set to 10 deg/s. Participants were familiarised with the control method in a single practice trial before the experiment.

#### Virtual reality system

**Fig 2:**
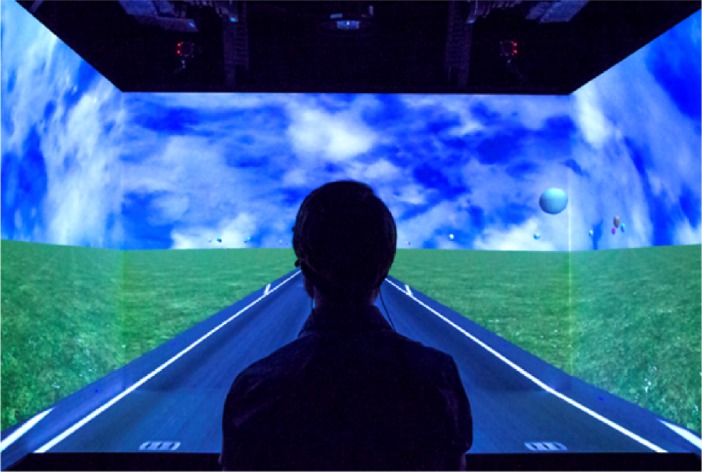
Depiction of one of the authors observing the visual environment in Experiment 1.

The virtual environment was rendered using a high end projection-based VR system (HoloStation, Christie Digital Systems Inc., Cypress, CA). The apparatus contained four projectors (Christie Mirage WU-L DLP®, resolution per projector: 1920 x 1200) that were controlled by a high-end computer (Z820, HP Inc., Palo Alto, CA) with two NVIDIA Quadro K6000 graphic cards and an NVIDIA G-SYNC (NVIDIA, Santa Clara, CA) card for frame synchronization. An optical motion capture system (4 x Trackpack, Advanced Realtime Tracking, Weilheim i.OB, Germany) tracked the position and orientation of markers mounted on stereo shutter glasses at a frequency of 120 Hz in order to couple position and orientation of the observer’s head to the projected view of the 3D environment. The same system was also used to track the hand-held controller. The projectors displayed the virtual environment on four screens: fronto-parallel, left, right and bottom screen (Fig 2). The fronto-parallel screen was 173 cm x 109 cm (width x height); the left and right screens were 108 cm x 109 cm; and the bottom screen was 173 cm x 108 cm. The stimulus was rendered at 120 FPS and was viewed stereoscopically using polarising LCD shutter glasses (Christie Digital Systems Inc.) with a 60 Hz asynchronous refresh rate for each eye. This setup allowed us to project the scene to a large part of the lateral and ventral peripheral field of view (FOV; approximately 90 deg vertical and 160 deg horizontal).

#### Design

The type of BCV experienced was designed as a between-subjects factor with three levels. In the control group, we applied no stimulation to the vestibular system. In the first experimental group we applied stimulation to the vestibular system when the angular acceleration of the projection camera reached a threshold (3 deg/s^2^). (From here we will refer to this as the ‘coupled group’, since BCV was coupled to the angular acceleration of the camera.) In the second experimental group we applied stimulation to the vestibular system at random intervals with an average frequency of occurrence of 0.9 Hz. We selected this frequency to match the occurrence frequency of stimulation for participants in the ‘coupled’ group during a pilot experiment. As a result, participants in this group received approximately 80 pulses of BCV during a single trial. (From here we will refer to this as the ‘random’ group; that is, BCV at random intervals). In this experiment, the number of vibration pulses experienced by participants in this group was not significantly different to 80 (one sample *t*-test, *p* = .29).

Whether or not participants were given control over their flight path was designed as a within-subjects factor with two levels. In half of the trials the participant actively navigated the path using the motion controller (we term this the ‘active’ condition). In the other half of the trials the participant traveled passively through the environment according to pre-recorded motion trajectories that we obtained from a pilot experiment (we term this the ‘passive’ condition).

#### Procedure

The participant entered the room and was told the goal of the task and instructed how to use the motion controller to navigate. The participant was seated on a chair such that the eyes of the participant were positioned approximately 148 cm from the fronto-parallel projection screen, and approximately 50 cm above the bottom screen. The experimenter positioned the bone vibrators on the skin at the mastoid processes and ensured symmetrical placement on both sides of the head. An elasticated headband was used to keep the vibrators stationary. At this stage the participant was presented with the standard magnitude of BCV, and the experimenter adjusted and recorded the magnitude if necessary.

A trial began with the presentation of a static view of the visual scene. The experimenter then pressed a button on the keyboard to commence the movement of the projection camera. Depending on whether the block contained ‘active’ or ‘passive’ trials, the participant would begin to navigate the path using the motion controller, or would begin to travel passively through the environment.

The experiment commenced with the practice trial, which lasted approximately two minutes. Following the practice, each trial lasted approximately 90 seconds. During the trial, BCV was either coupled with angular accelerations of the camera (‘coupled’ condition), applied randomly at 0.9 Hz average frequency of occurrence (‘random’ condition), or was absent (‘control’ condition) based on the random group assignment of the participant. A target disappeared if the projection camera came within 0.5 m of the edge of the target. Once the path was complete, the experimental program terminated. Trials where the participant missed targets were not repeated, given that doing otherwise would have required an increased duration of exposure to the VR conditions compared to other participants. Data obtained following these trials were included in analyses. For each of the two levels of movement control (active or passive) we presented 5 trials. Each of the passive trials adhered to a different pre-recorded motion trajectory, and the order of these was uniquely randomized for each participant. This resulted in a total of 10 trials per participant. We blocked the design of the study by the type of trial (active or passive). Half of the participants experienced active trials first and the other half experienced passive trials first. There was a 5 minute break included following the first block of trials, after which the participant commenced the second block.

Participants completed a Simulator Sickness Questionnaire (SSQ) after every trial. This involved a checklist of 16 symptoms such as ‘nausea’, ‘fatigue’, and ‘headache’. For each item on the checklist, we asked participants to indicate the amount to which they currently experienced that symptom using the options ‘none’, ‘slight’, ‘moderate’, or ‘severe’. The experiment lasted approximately 45 minutes to 1 hour in total including introduction and debriefing.

#### Data analysis

After the experiment the responses for items on the SSQ were used to compute a total SSQ score according to the guidelines of Kennedy and colleagues [1]. This total score exhibited a high degree of variability which was non-homogeneously distributed across groups, and as such we conducted a square root transformation on the data which resulted in homogeneity of variance. These transformed data were subjected to statistical analyses, as in Experiment 1.

We characterized the number of ‘sick’ participants in each condition by calculating average SSQ score across trials in a block and classifying ‘sickness’ as an average score of 20 or higher [55].

### Results

Participants displayed a high level of performance on the task. Out of the 30 participants who completed the study, 27 completed the task without missing a target, while the remainder missed an average of 3 targets across the 10 trials. Of the four participants who elected to terminate the experiment early due to high simulator sickness, two were from the random group, one was from the coupled group, and one was from the control group.

The most commonly reported symptom across all groups was ‘fatigue’ (percentage of participants who reported the symptom at least once: 93%). The next most common symptoms were ‘difficulty concentrating’ in the coupled group (73%), and ‘general discomfort’ in the random and control groups (73% and 83% respectively).

**Fig 3:**
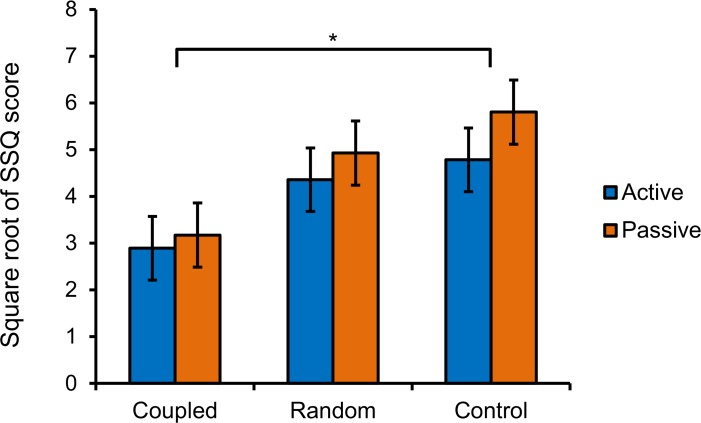
Experiment 1, square-root transformed SSQ for participants in different movement control and stimulation conditions. Error bars represent standard error of the mean. * *p* < .05.

We ran a mixed-factor 2 X 3 analysis of variance (ANOVA) on transformed SSQ scores for the within subjects factor of movement control (active or passive) and the between subjects factor of stimulation type (coupled, random, or none). Results revealed a main effect of stimulation type on the transformed SSQ scores, *F*(2, 27) = 3.46, *p* = .046, η^2^_*p*_ = 0.20 (Fig 3).We conducted a follow-up analysis using estimated marginal means on the factor stimulation type. The results showed that coupled vibration trials were associated with significantly lower transformed SSQ scores than control trials (*p* = .017). However, transformed SSQ scores in the random trials did not differ from those in the coupled trials (*p* = .08) or the control trials (*p* = .47). We found no main effect of movement control, *F*(1, 27) = 3.86, *p* = .06, η^2^_*p*_ = 0.13, although the active condition was related to slightly lower transformed SSQ scores than the passive condition.

There was no interaction between the factors stimulation type and movement control, *F*(2, 27) = 1.70, *p* = .20, η^2^_*p*_ = 0.11.

**Fig 4:**
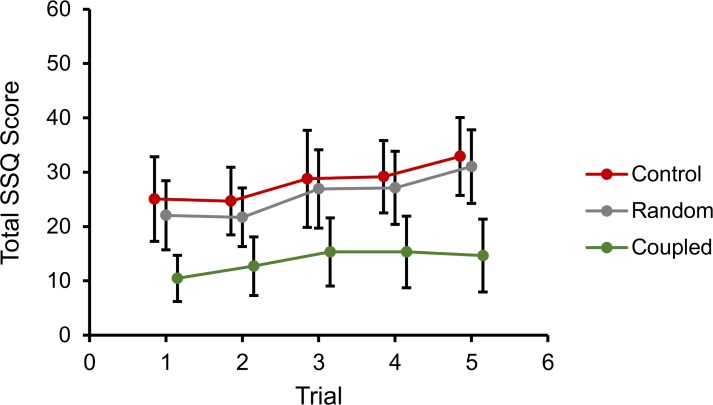
Experiment 1, total SSQ scores over trials for each stimulation condition. Error bars represent standard error of the mean.

To establish the degree to which simulator sickness increased across trials, we calculated linear trends for each group. We found a significant overall linear increase in the transformed SSQ scores over the five trials in a block, *F*(1, 27) = 5.41, *p* = .028, η^2^_*p*_ = 0.17 (Fig 4). This linear trend did not differ as a function of the levels of stimulation type (*F*(2, 27) = 0.51, *p* = .61, η^2^_*p*_ = 0.05) or movement control (*F*(1, 27) = 0.04, *p* = .84, η^2^_*p*_ = 0.01).

**Fig 5:**
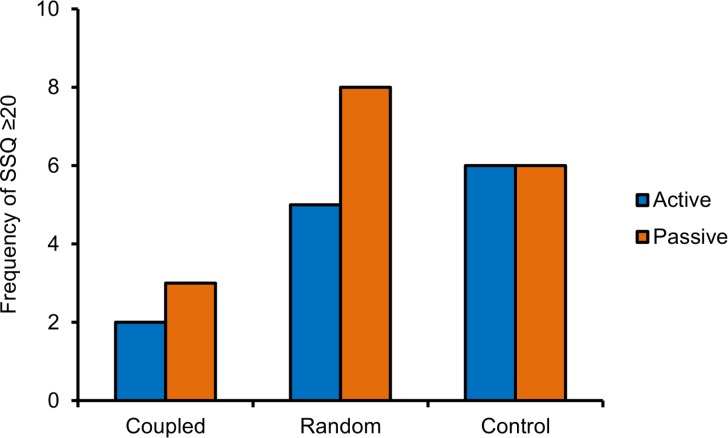
Experiment 1, number of participants classified as ‘sick’ in each condition. ‘Sickness’ corresponds to average SSQ scores ≥ 20 [55].

The number of participants who were classified as ‘sick’ in each condition is presented in Fig 5. The frequency of ‘sick’ participants was highest for the control group, second highest for the random group, and lowest for the coupled vibration group.

### Discussion

The data revealed an effect of vestibular stimulation on simulator sickness scores. A follow-up analysis showed that transformed simulator sickness scores were lower when BCV was coupled with large angular accelerations of the projection camera. Our initial hypothesis was supported, in that the effectiveness of noisy vestibular stimulation imparted a benefit when it was applied concurrently with expected vestibular signals. The results show that comfort in a high-end virtual reality experience can be improved by the application of noisy vestibular stimulation, which is relatively cheap, non-invasive, and easy to use.

The results show that the type of movement control used had little effect on simulator sickness scores. The degree of vestibular stimulation applied had much more of an impact on the pattern of data we obtained. We note that multiple studies have reported that passive movement tends to produce higher simulator sickness than active movement [49–51]. We were unable to corroborate these findings. However, it is possible that this result could be related to differences in movement variability between the passive and active trials that we discuss in detail below (see General discussion).

The data support the idea that simulator sickness can be reduced through the use of noisy vestibular stimulation in a high-end, projection based virtual reality system. However, most users of VR do not have access to such high performance equipment. On the other hand, the use of head-mounted displays is becoming widespread with the release of technology such as the Oculus Rift, the HTC Vive, and the FOVE head-mounted display. This hardware can be purchased at a low-cost and maintains good standards in terms of display refresh rate, head tracking, and motion-to-photon latency. At the same time, devices such as the Oculus Rift are known to produce simulator sickness [56]. Our next question was about the degree to which the stimulation technique used in Experiment 1 reduces simulator sickness in visual display conditions that are more relevant to consumer practices. In order to answer this question we conducted Experiment 2 where we closely replicated the task of Experiment 1 using an off-the-shelf head-mounted display, and applied noisy vestibular stimulation during the task.

## Experiment 2

### Methods

#### Participants

Participants were recruited from a student mailing list and an undergraduate Psychology course credit pool at Queen’s University. There were 78 participants (51 women) who completed the experiment. As in Experiment 1, sample size was selected a-priori and participants who terminated the study early were replaced until the desired sample size was met. One participant terminated the experiment early due to a high level of simulator sickness, and her data were not included in the final analyses. We compensated each participant $10 per hour, or 1 course credit per hour for an undergraduate psychology course. Mean age was 18.18 years (*SD* = 0.66, range = [17, 21]). All participants had normal or corrected to normal vision: Corrective glasses were used in the head-mounted display if they were required by the participant.

We screened participants for motion sickness susceptibility similarly to Experiment 1 (rated from 0 to 10), and as in Experiment 1 we received no scores that required the participant to be excluded (maximum score of 7).

#### Visual stimulus

**Fig 6:**
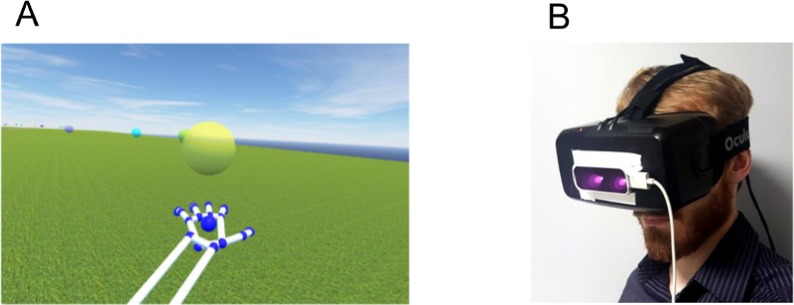
Virtual environment in Experiment 2. A) The virtual environment seen by participants. B) The head-mounted display that was used to visualise the environment. The Leap Motion Controller was mounted on the front of the display device.

The visual stimulus was rendered using the Oculus software developer kit (version 0.8.0) and Oculus plugin in Unity3D (version 5.0; Unity Technologies SF, San Francisco, CA). The internal head tracking of the device (translation and rotation) was used to update the viewpoint of the observer, so that the virtual environment appeared to be stable. The stimulus was designed such that it appeared as visually identical as possible to the task in Experiment 1 (that is, a 350 by 100 meters grassy plane; a 350 by 5 meter runway for visual reference; 30 target spheres positioned at points along a sinusoidal specified as: *y* = 40 sin(2 π *x*/157.5), where *y* is left-right and *x* is fore-aft; a target diameter of 1 metre and a target height of 3 metres above the ground plane; Fig 6).

Although the virtual environment was the same in both experiments, participants in Experiment 1 had the ability to see their arm and hand while controlling movement of the projection camera. For participants using the head-mounted display in Experiment 2, this was not possible. Therefore, in Experiment 2 we asked participants to hold their left hand in front of their body, and motion captured the position and orientation of the hand. We projected a visual representation of the hand into the virtual environment (Fig 6A). In addition, we used the rotation of the left hand to control the heading of the projection camera. Motion capture of the hand was achieved using a low-cost infrared hand-tracking camera (Leap Motion Controller, Version 3.0.0, Leap Motion Inc., San Francisco, CA) and the pre-built Unity Asset Package. The hand and forearm were visually represented by a skeleton that was included in the Unity Asset Package for the hand-tracking camera.

The projection camera maintained a constant velocity of 5.5 m/s in the direction of heading, and the heading direction was controlled by the orientation of their left hand. Note that, similarly to Experiment 1, heading was de-coupled from gaze direction such that the participant could rotate their head while maintaining their heading direction. The rotation angle of the hand in pitch, roll, and yaw in world coordinates was used to set the angular velocity of the camera in each axis (as in Experiment 1, the orientation of the hand in degrees defined angular velocity in degrees per second for pitch, roll, and yaw).

#### Virtual reality system

The virtual environment was rendered using a low-cost head-mounted display running at 75 FPS (Oculus Rift DK2; Oculus VR, Menlo Park, CA; resolution per eye: 960 x 1080). The presentation of the virtual environment was delivered by a high-end graphics computer (HP Z820; NVIDIA Quadro K6000 graphic card).

#### Design

The experimental design reproduced Experiment 1. The type of BCV was a between-groups factor (conditions were identical to Experiment 1: ‘Coupled’, vibration applied with 3 deg/s^2^ angular acceleration of the camera; ‘Random’, applied at 0.9 Hz; or ‘Control’, no vibration). Movement control was a within-subjects factor with two levels (‘active’ or ‘passive’; hand-controlled navigation or automatic navigation, respectively).

#### Procedure

The participant entered the room and was introduced to the goal of the task and instructed how to use their hand to navigate. The participant was seated on a chair approximately 50 cm in front of the positional tracking camera of the head-mounted display, and approximately 100 cm above the ground. The experimenter positioned the bone vibrators on the skin at the mastoid processes and ensured symmetrical placement on both sides of the head. An elasticated headband was used to keep the vibrators stationary. At this stage the participant was presented with the standard magnitude of BCV, and the experimenter adjusted and recorded the magnitude if necessary.

The task progression was similar to Experiment 1. Before the experiment, participants were familiarised with the control method in a single practice trial which lasted approximately two minutes. Trials began with the presentation of a static view of the visual scene. The experimenter then pressed a button on the keyboard to commence the movement of the projection camera. Depending on whether the block was an ‘active’ or ‘passive’ trials, the participant would begin to navigate the path by rotating their hand, or would begin to travel passively through the environment. Each of the passive trials adhered to a different pre-recorded motion trajectory, and the order of these was uniquely randomized for each participant. Each participant completed 10 trials (blocks of 5 active and 5 passive, the order of which was counterbalanced).

Each trial lasted approximately 90 seconds. During the trial, BCV was applied based on the random group assignment of the participant. A target disappeared if the projection camera came within 0.5 m of the edge of the target. Once the participant had completed the path, the experimental program terminated. Participants then removed the head mounted display and completed the SSQ. Trials involving missed targets were not repeated and data following these trials were included in statistical analyses. There was a 5 minute break following the first block of trials, after which the participant commenced the second trial block. The experiment lasted approximately 45 minutes to 1 hour in total including introduction and debriefing.

#### Data analysis

As in Experiment 1, responses for items on the SSQ were used to compute a SSQ total score. We conducted a square root transformation on the data to correct for heterogeneity of variance before statistical analyses were performed. These transformed data were subjected to statistical analyses. We again grouped participants as ‘sick’ or ‘not sick’ using the criterion of 20 or higher for the average total SSQ score across trials in a block [55].

### Results

The majority of participants completed the experiment without any tracking failures. Of the 78 participants who completed the experiment, 45 did so without missing a target, while the remainder missed an average of 2 targets across the 10 trials. Most of these misses were produced when the motion capture system momentarily failed to track the orientation of the hand. The participant who elected to terminate the experiment early due to high simulator sickness was from the coupled group. A one-sample *t*-test confirmed that the frequency of vibration pulses in the coupled group across the two experiments was similar (*p* = .11).

The most commonly reported symptoms across all groups were ‘fatigue’ and ‘general discomfort’ (percentage of participants who reported the symptom at least once was 85% and 72% respectively). The next most common symptom was ‘dizziness eyes closed’ in the coupled group (64% of participants), ‘headache’ in the random group (67% of participants), and ‘eyestrain’ in the control group (71% of participants).

**Fig 7:**
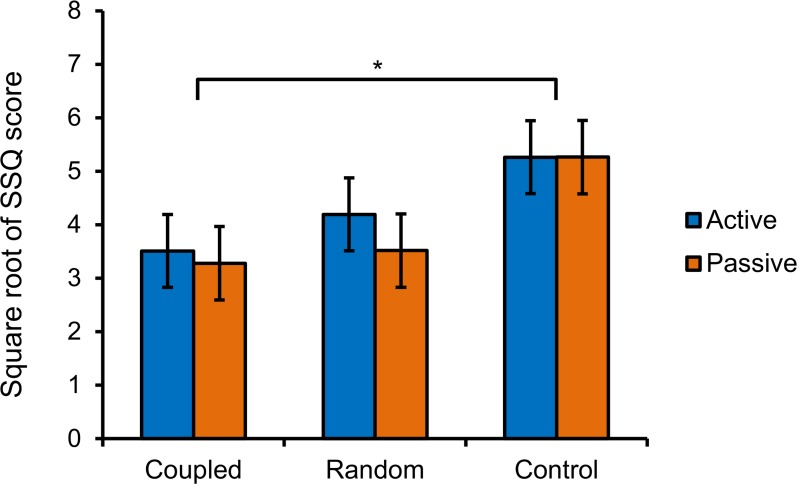
Experiment 2, square-root transformed SSQ for participants in different movement control and stimulation conditions. Error bars represent standard error of the mean. * *p* < .05.

We ran a mixed-factor 2 X 3 ANOVA on SSQ scores for the within subjects factor of movement control (active or passive) and the between subjects factor of stimulation type (coupled, random, or control). We observed a main effect of stimulation type on transformed SSQ scores, *F*(2, 75) = 3.59, *p* = .033, η^2^_*p*_ = 0.09 (Fig 7) and conducted a follow-up analysis using estimated marginal means on the factor stimulation type. The results showed that coupled trials were associated with significantly lower transformed SSQ scores than control trials (*p* = .012). Transformed SSQ scores in the random trials did not differ from those in the coupled trials (*p* = .06) or the control trials (*p* = .53).

We found no effect of movement control on transformed SSQ scores, *F*(1, 75) = 2.73, *p* = .10, η^2^_*p*_ = 0.03, which replicated the result of Experiment 1 where the difference between active and passive trials was also non-significant.

There was no interaction between the factors of stimulation type and movement control, *F*(2,75) = 1.20, *p* = .31, η^2^_*p*_ = 0.03.

**Fig 8:**
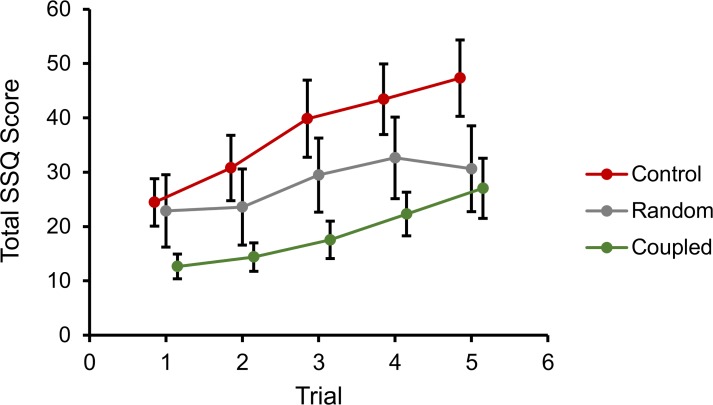
Experiment 2, total SSQ scores over trials for each stimulation condition. Error bars represent standard error of the mean.

To establish the degree to which simulator sickness increased across trials, we calculated linear trends for each group. We found a significant linear increase in transformed SSQ scores over the five trials in a block, *F*(1, 75) = 46.29, *p* < .001, η^2^_*p*_ = 0.39 (Fig 8). This linear trend did not differ as a function of the levels of stimulation type (*F*(2, 75) = 0.63, *p* = .94, η^2^_*p*_ = 0.02) or movement control (*F*(2, 75) = 1.30, *p* = .26, η^2^_*p*_ = 0.02).

**Fig 9:**
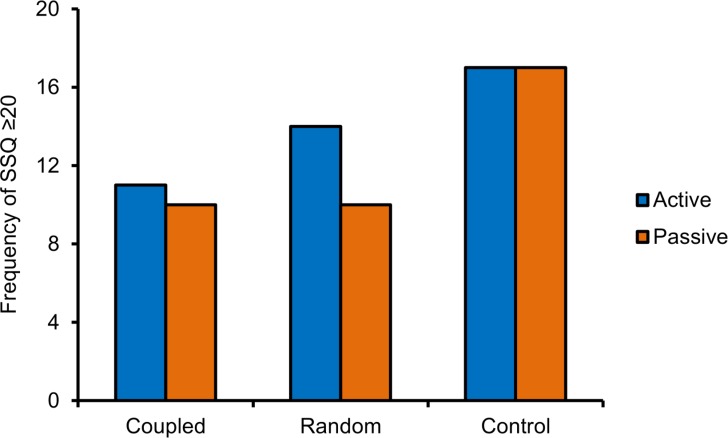
Experiment 2, number of participants classified as ‘sick’ in each condition. ‘Sickness’ corresponds to average SSQ scores ≥ 20 [55].

As in Experiment 1, the number of participants who were classified as ‘sick’ (SSQ scores of 20 or higher) was highest in the control group, second highest for the random vibration group, and lowest for the coupled vibration group. The data are presented in Fig 9.

### Discussion

Similarly to Experiment 1, the data supported our hypothesis that noisy vestibular stimulation can influence simulator sickness in VR if it is applied when vestibular signals are expected (i.e., coupled with large visual acceleration). As well, we found no dependency of simulator sickness on the type of movement control used, as in Experiment 1. Although the effect of coupled BCV was small (Exp. 1, η^2^_*p*_ = 0.20; Exp. 2, η^2^_*p*_ = 0.09), the fact that we obtained the result in both experiments suggests this may be a reliable effect.

## General discussion

### Comparison of display conditions

We tested the effect of noisy vestibular stimulation on simulator sickness scores for a large field-of-view screen-projected VR system (Christie Holostation) and an off-the-shelf head-mounted display (Oculus DK2). In general we observed a subtle, yet statistically significant effect of stimulation that was similar across the two display conditions. In both Experiments 1 and 2, participants exhibited less simulator sickness in the condition where vibration was coupled with angular accelerations of the camera compared with control (fewer than half experienced SSQ scores of 20 or above). Control conditions in both experiments evoked a large degree of simulator sickness on average (more than half experienced SSQ scores of 20 or above). And, in both cases vibration applied at random intervals was not statistically different to either the coupled or control groups (approximately half of participants in this group experienced SSQ scores of 20 or above).

While the current study was not designed to compare the difference in symptom severity between the display conditions, it is worth noting that the average outcome measures tended to be similar between Experiment 1 and 2. The results also provide evidence that the vestibular stimulation we applied here generalizes well across display conditions. By replicating the task between the two experiments, we gained a first exploratory insight about the possible modulation of the effect of BCV on simulator sickness by any of the factors that varied between the two conditions (e.g., refresh rate, field-of-view). The fact that the effect was replicated suggests that the technique has potential for use in generalized VR display conditions.

### Noisy vestibular stimulation and simulator sickness

We observed that transformed SSQ scores were significantly lower for participants who received coupled vestibular stimulation compared to control, and we found a non-significant tendency for scores in the coupled stimulation group to be lower than scores in the random stimulation group. Therefore, the results provide some limited support for our theory that noisy vestibular stimulation reduces the weight of vestibular signals through a process of changing vestibular sensory reliability [44]. Building on the sensory conflict account of simulator sickness [13], our motivation was to manipulate relative cue reliability between vestibular and visual cues and encourage visual cues to dominate self-motion perception. However, we did not measure sensory conflict in our experiments, and cannot rule out the possibility of other influences that we did not measure here (e.g., postural instability [18–21]).

Significant trends obtained in Experiments 1 and 2 indicated that simulator sickness scores tended to increase linearly over blocks of trials. We did not find any interaction between this increase and the type of stimulation used. This suggests that BCV coupled with angular acceleration may be effective for reducing the average simulator sickness experienced by a participant, but that the rate of increase in symptom severity is unaffected. Therefore, while long sessions of VR exposure may still lead to intolerable levels of simulator sickness, the technique described here might affect the length of time for which a user can tolerate VR.

The effect of coupled BCV on simulator sickness was small, particularly in Experiment 2. While the technique might be improved and refined in the future (see discussion below), the small effects we observed may limit the practical appeal of the method for improving comfort in VR. This is especially true given that the current method involves careful initial adjustment of the location of the bone vibration devices, which is undoubtedly impractical for use with VR when the purpose is entertainment. On the other hand, there may be practical utility to such a technique in settings outside of consumer entertainment, where learning and task performance can be diminished by simulator sickness (e.g., training of pilots in flight simulations)[3].

### Passive and active movement control

Our data indicate that the degree of simulator sickness experienced was relatively similar regardless of whether or not the participants had control over the motion trajectory of the projection camera. We were unable to provide support for the results of previous literature that has linked control of movement to the likelihood of experiencing simulator sickness [49–51]. Anecdotal reports from the research assistants suggest that the active trials tended to exhibit higher variability in heading direction than the trajectories we used for the passive trials. We noted that although the passive trajectories contained a large amount of variability in their motion trajectory, they did not reach the extreme variability experienced by some participants who had difficulty maintaining a smooth course during the navigation task. In future experiments it would be interesting to yoke the movement of one participant (active) to another participant (passive), as in other studies [49–50] so as to maintain a constant level of variability in both the active and passive conditions. As well, there might have been an additional increase to simulator sickness scores in active trials caused by hand-motion tracking problems. Particularly in Experiment 2, there were several occasions where the hand tracking of participants failed momentarily, causing the camera to be unresponsive to movements.

Overall, the fact that simulator sickness was reduced equally for active and passive conditions adds to the promise of the technique used here. VR applications such as driving and flight simulation tend to include passive motion that often leads to increased symptom severity [49–51]. Other types of passive movement that are associated with a high degree of simulator sickness (such as simulated walking with head bob) should be studied in order to assess the task-generalizability of the effect we observed here.

### Future direction

The findings present the possibility that BCV could operate as a cheap and effective way to alleviate or even prevent simulator sickness in VR. However, significant further testing will need to be carried out on this technique given both the novelty and relatively specific set of parameters tested so far. We find it encouraging that we observed an effect of BCV on simulator sickness, even though our method of coupling stimulation to the angular acceleration of the camera was in some ways arbitrary. Our general rationale was that angular accelerations are one of the primary stimuli for the vestibular system, and as such we added vestibular noise at these critical times when significant vestibular cues should be expected to occur. We did not include a condition where vibration was coupled to linear accelerations, which are equally adequate for vestibular stimulation. It would also have been possible to apply BCV continuously, given that tilt is a constant source of information used by the vestibular system to judge head orientation. Constant BCV stimulation during VR use might be too unpleasant or intrusive for many practical applications. Nonetheless, it would be useful to conduct a similar experiment in the future that contains the addition of a constant vibration condition. Such a study would add to our understanding of whether coupled stimulation is a necessary aspect of the use of BCV to reduce simulator sickness. There could also be much to learn from an experiment that employs BCV for an extended duration prior to–but not during–exposure to VR. It may be reasonable to assume that there are both short term effects of BCV, as we have documented here, and sustained effects on sensory reweighting and simulator sickness that can be observed over a longer period. This study marks the first test of BCV to approach simulator sickness reduction in VR, and we are positive that variations of the technique could show large improvements over the subtle effects we observed here.

Our conclusions about the effect of BCV on sickness symptoms are qualified by the fact that transformed SSQ scores in the random group did not differ from scores in the control or coupled groups. At this point, we are limited to speculation about why the random group was not different to either group. Given an increased experimental duration, we might have observed a difference between the random and control groups. The result could suggest that noisy vestibular stimulation reduces simulator sickness irrespective of the nature of stimulation. This finding again shows the need for future, high-powered studies intended to replicate the effects observed here.

The various aspects of the technique that we did not manipulate here include the duration of stimulation, the frequency and magnitude of the vibration, and the way it is coupled to the different kind of vestibular stimulation which the brain might expect in a simulated environment. Given that the task was the same in both experiments, we cannot state whether the effect observed here would generalize to other tasks (e.g., Does the effect of BCV generalize to driving simulators?) or more nauseogenic conditions (e.g., Does the effect persist when optic flow speed is much higher?). Similar questions surround the individual differences in the effectiveness of stimulation (e.g., Why might some participants benefit from coupled BCV, while others might not?). We provided evidence that the effect of stimulation generalized across two display conditions, but there are a multitude of other VR display technologies that may or may not benefit from this technique. Another aspect that is crucial to investigate is the degree to which the vibration can be presented unobtrusively (e.g., utilizing vibration frequency which is inaudible), or embedded within another auditory stream. Such a method might reduce the amount of distraction caused by the BCV technique–certainly, all participants in our study noticed the vibration that was applied, and this might reduce immersion into the VR experience. While the technique might prove useful in training or rehabilitation settings where skills acquisition is limited by simulator sickness, the issue of intrusiveness undoubtedly limits the use of BCV in VR entertainment experiences. Finally, the degree to which this technique could be applied to reducing motion sickness outside of VR is unknown. Since both simulator sickness and motion sickness are likely related to sensory mismatches [12–13], the technique might also prove effective in reducing the severity of common symptoms of travel sickness and sea sickness. Resolving these open questions will move the field closer to the development of a consumer-oriented therapy for sensory mismatch-induced sickness that can be used in a broad range of conditions.

### Conclusion

We have outlined evidence from two experiments showing that simulator sickness in virtual reality can be reduced by time-coupling BCV to the occurrence of visual angular accelerations. At the same time, we found no difference between conditions where vibration was random or time-coupled to angular accelerations. While the effects we obtained were small, the current study constitutes the first research to implement this technique with the aim of reducing the negative side-effects of VR experiences. More research will be needed to determine if refinements to the technique will result in increased effects. Future experiments should also assess factors such as the effect of long-term BCV application on simulator sickness, how individual differences modulate the effectiveness of the technique, and whether the intrusiveness of application can be reduced while retaining (and improving) efficacy.
